# Supplementary material for: A Pumpless Microfluidic Neonatal Lung Assist Device for Support of Preterm Neonates in Respiratory Distress
Source: Adv Sci (Weinh). 2020 Sep 29;7(21):2001860. doi: 10.1002/advs.202001860 (PMC7610273; doi:10.1002/advs.202001860)
Supplement: Supplementary file 1 — Supporting Information [file ADVS-7-2001860-s001.pdf]

Supplementary Information

A Pumpless Microfluidic Neonatal Lung Assist Device for Support of Preterm Neonates in Respiratory Distress

Mohammadhossein Dabaghi[1], Niels Rochow[2,6], Neda Saraei[3], Gerhard Fusch[2], Shelley Monkman[2], Kevin Da[4], Alireza Shahin-Shamsabadi[1], John L. Brash[1,4], Dragos Predescu[2], Kathleen Delaney[5], Christoph Fusch[1,2,6,] and P. Ravi Selvaganapathy[1,3]

[1]School of Biomedical Engineering, [2]Department of Pediatrics, [3]Department of Mechanical Engineering, [4]Department of Chemical Engineering, [5]Central Animal Facility Department McMaster University, Hamilton, ON, Canada, [6]Paracelsus Medical University, Nuremberg, Department of Pediatrics, University Hospital Nuremberg, Germany

In vitro carbon dioxide release for LAD

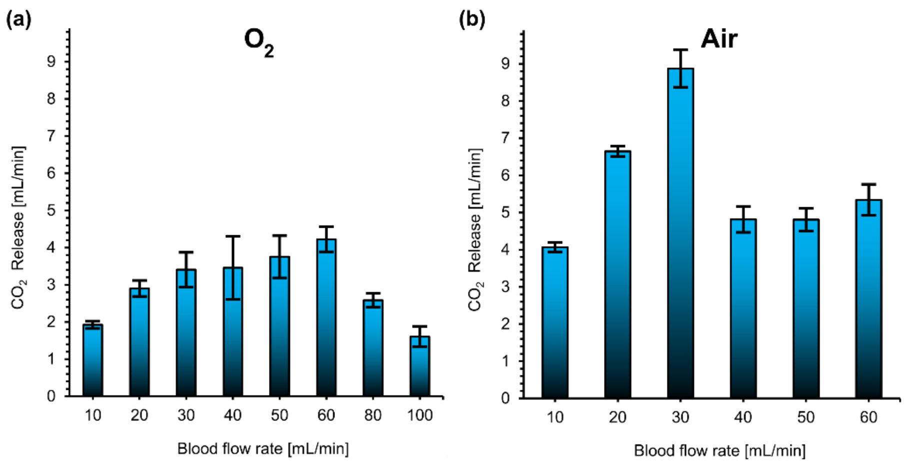

Figure S1: (a) CO_2 release at various blood flow rates while the LAD was consuming oxygen as the sweep gas and (b) CO_2 release at various blood flow rates while the LAD was exposed to room air.

The sequence of conditions for in vivo study

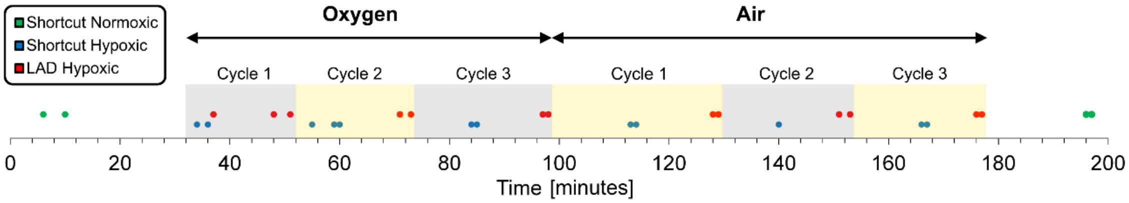

Figure S2: Time sequence of in-vivo measurements indicating the conditions that the piglet was exposed to and the time points when blood samples were extracted. The LAD was tested first with pure oxygen and then switched to room air. The x-axis represents the time and points represents when blood samples were taken with the alternating background color for cycle changes.

Access to the right atrium of the heart

A 3.5 Fr Argyle umbilical catheter was used to access the right atrium of the heart via the right internal jugular vein. To guide the catheter to the designated location and ensure that the catheter is not clogged, ultrasonography was used.

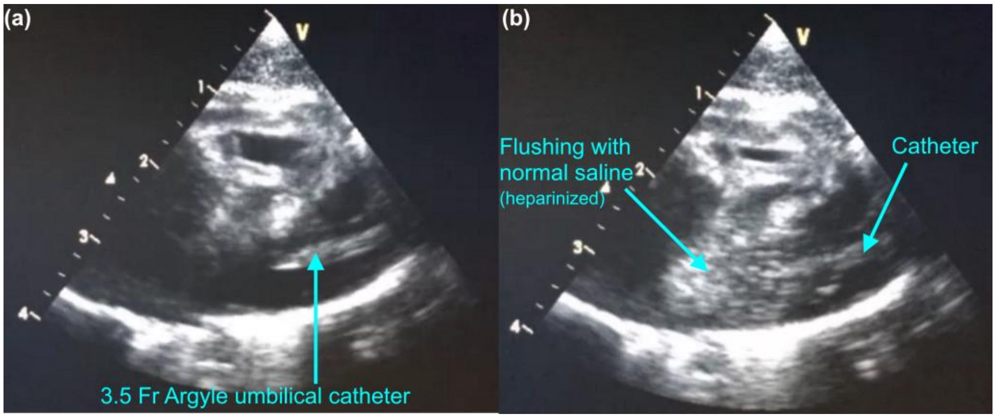

Figure S3: Ultrasonography of the right atrium of the heart: (a) securing a 3.5 Fr Argyle umbilical catheter and (b) flushing the line with heparinized normal saline solution.

Figure S3a shows that the catheter was placed in the right atrium of the heart. And it was flushed with heparinized normal saline showing that it was not clogged (Figure S3b).

Achieved blood flow rates and pressure drops over time

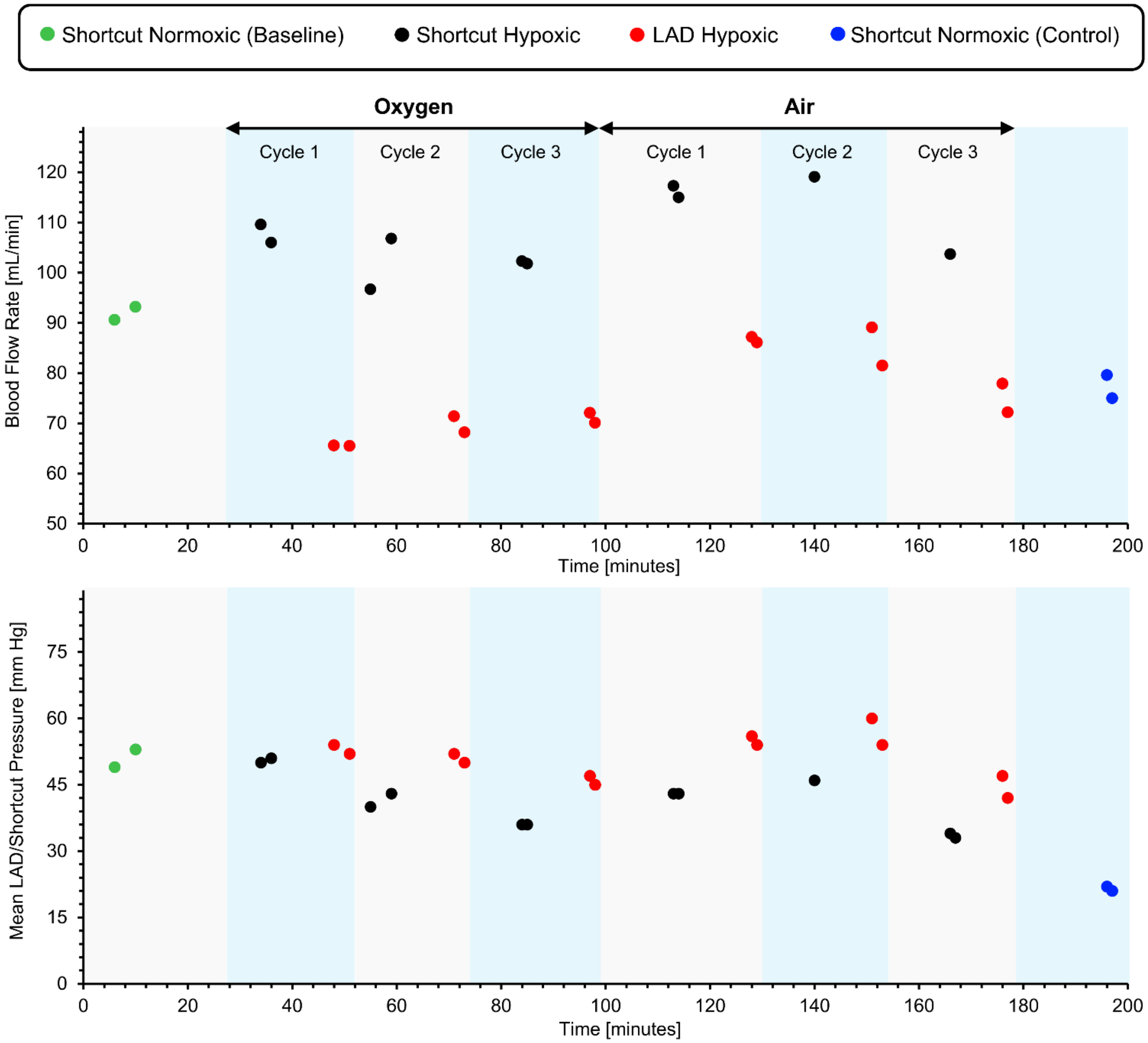

Figure S4: (a) measured blood flow rates over the period of the experiment and (b) the shortcut or the LAD's pressure during the experiment.

Systemic oxygen saturation measured by pulse oximetry

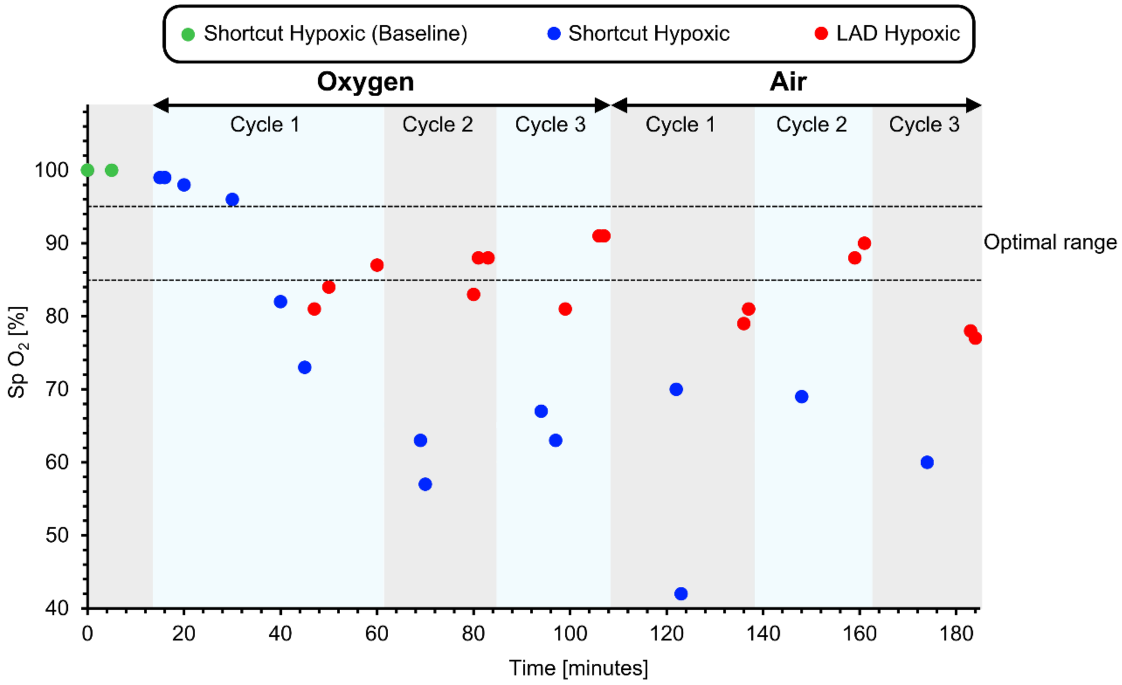

Figure S5: systemic oxygen saturation level measured by a pulse oximeter placed on the left foot.

Metabolic analysis by sampling from the femoral artery

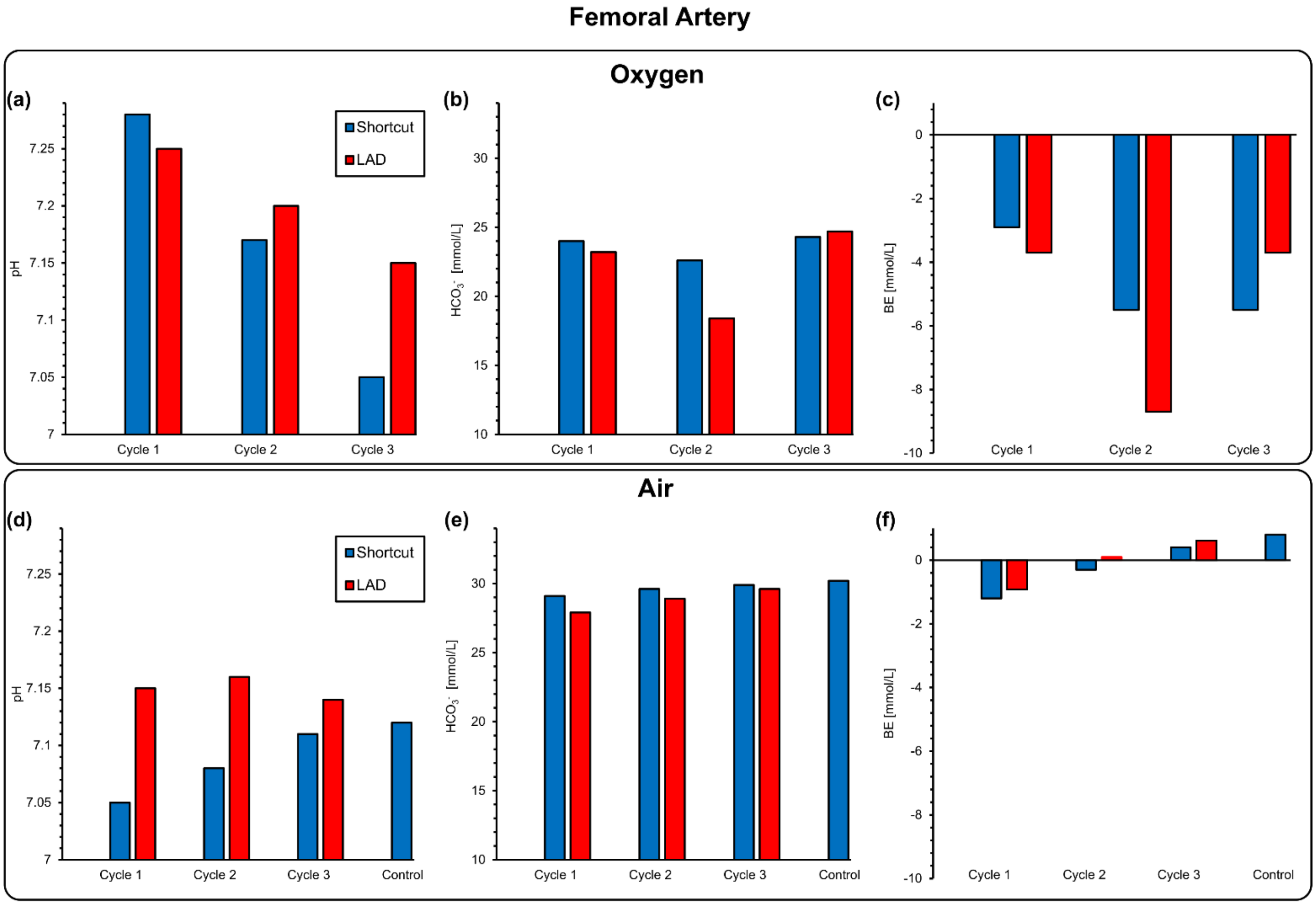

Figure S6: The metabolic analysis: (a) pH measurement, (b) bicarbonate content, (c) base excess pCO_2 for femoral artery using oxygen as the sweep gas, and (d) pH measurement, (e) bicarbonate content, and (f) base excess for femoral artery exposed directly to room air.

In vitro comparison between LADs with different channel heights

Since the LAD was supposed to be operated without the need of a pump, we used a numerical model that we introduced in our previous works[1,2] to estimate a range for the height of MBOs so that the LAD could operate in a pumpless manner. For a newborn piglet weighing 1.5 – 2 kg, we assumed that a blood flow rate of 60 – 90 mL/min would be enough to provide respiratory support for the piglet under a hypoxic condition. At a flow rate of 60 – 90 mL/min, the pressure drops of blood flow distributors (Figure S9a), tubing, connectors, and catheters were calculated to be 35 – 45 mm Hg in total. We assumed that the maximum pressure differential provided by the heart of the animal would be 60 mm Hg (based on our previous experience[3]). Therefore, MBO's pressure drops had to approximately range from 15 mm Hg to 25 mm Hg that would generate an overall pressure drop of ~ 60 mm Hg for the whole circuit and the LAD. Considering the pressure drop range and the overall blood flow rate of 60 – 90 mL/min, which would lead to a blood flow rate range of ~ 4 – 6 mL/min for MBOs assuming that the LAD had 16 MBOs, the height of MBO had to be somewhere between ~ 130 µm and ~ 180 µm. Although it was expected to see the LAD made of devices with channels' height of 130 μm would perform better, we did not see that such a big difference. In our previous works[1,2,4,5] that we introduced several versions of double-sided microfluidics blood oxygenators, we observed a similar trend that the oxygenation of such a device would not be much compromised by increasing the height of the blood channels. Oxygen molecules on their way to reach red blood cells, they should first diffuse through the membrane, then dissolve in the blood and diffuse through plasma. Our previous results[2,5] suggested that the oxygen transfer did not change significantly in a range of the channels' height due to the fact that oxygen transfer was mainly controlled by diffusion in plasma. Therefore, we took advantage of this phenomenon and fabricated MBOs with taller height to reduce the pressure drops.

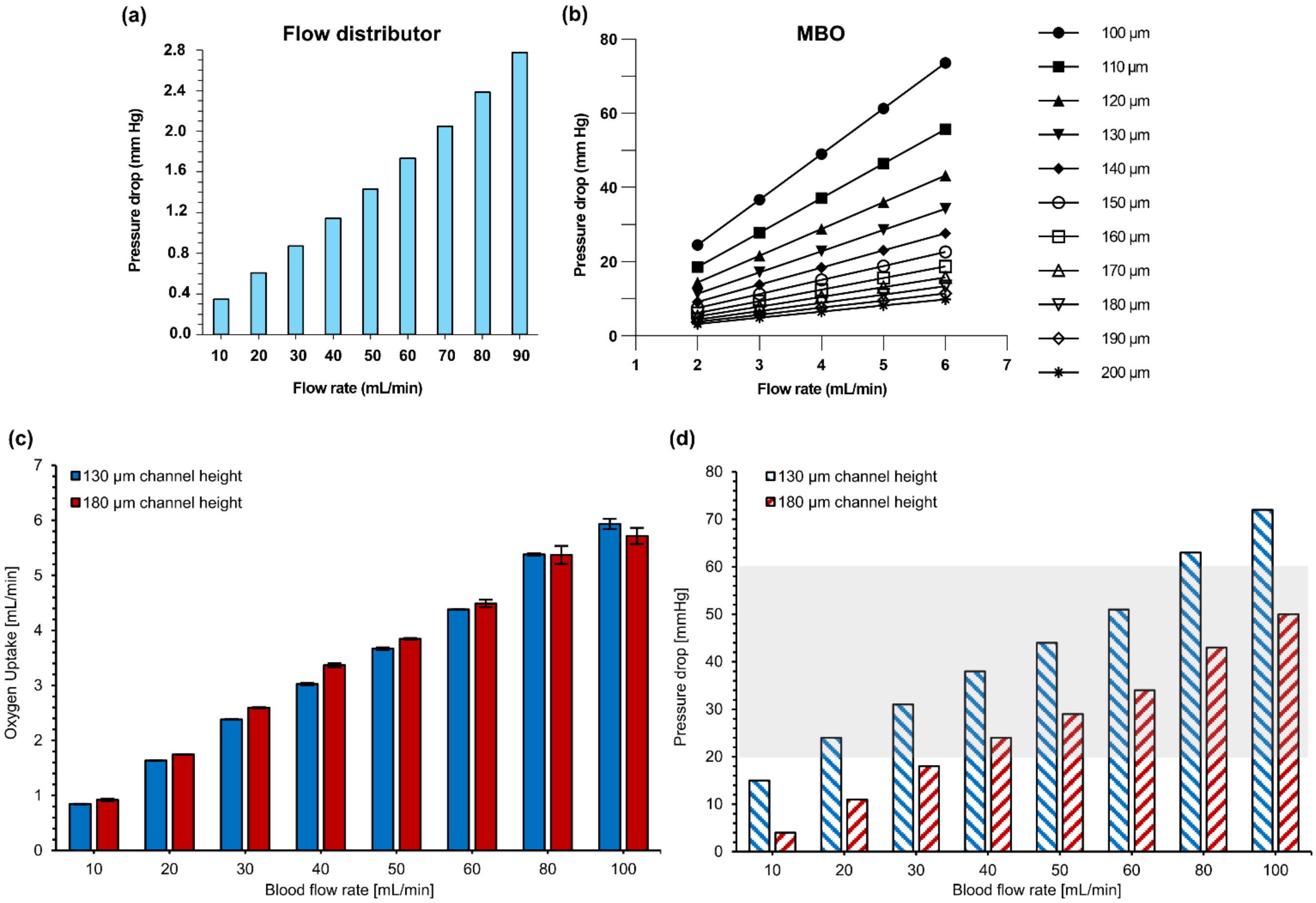

Figure S7: (a) the numerical results of pressure drop for the flow distributor versus blood flow rates, (b) the numerical results of pressure drops for MBO versus blood flow rates, in vitro comparison between LADs with two different microchannel heights of 130 μm and 180 μm with respect to blood flow rate: (c) oxygen uptake in an oxygen-rich environment and (d) pressure drop (the shaded gray region represents the operating pressure drop range for preterm infants).

Cardiovascular parameters for the LAD with a microchannel height of 130 µm

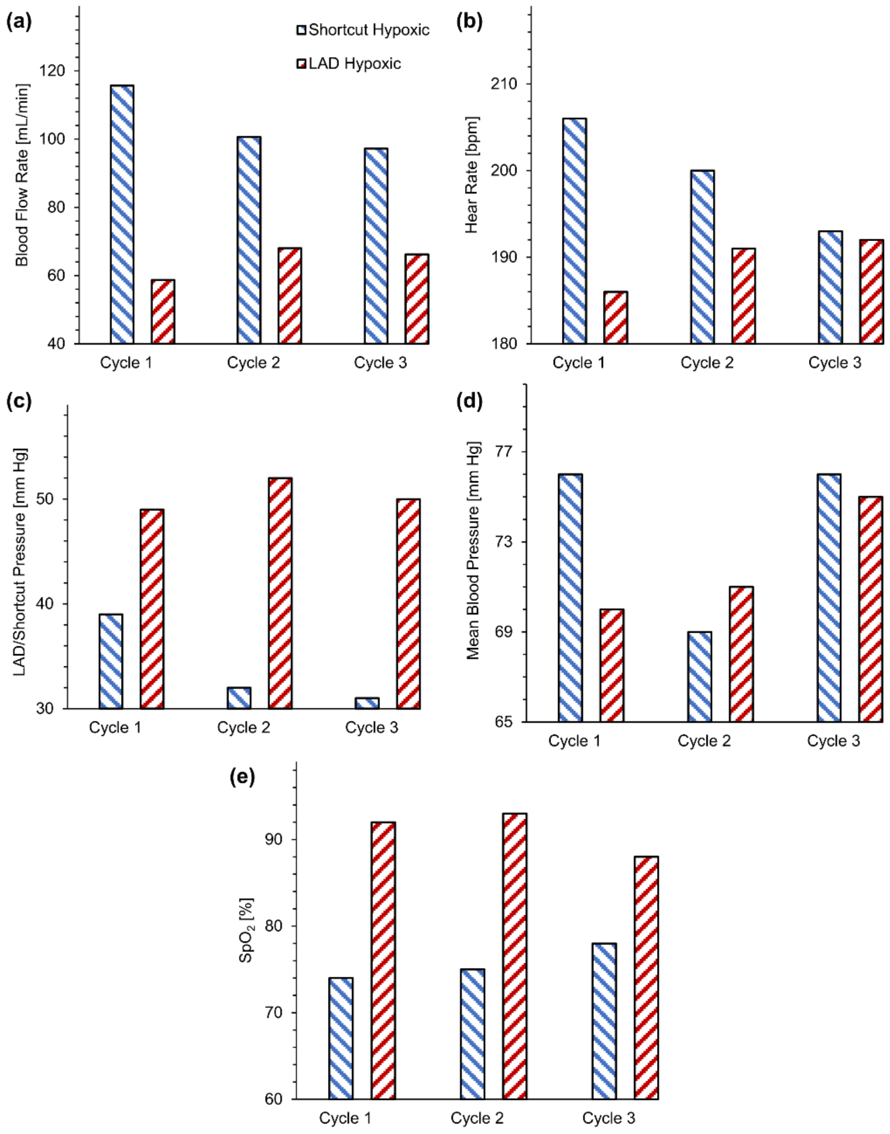

Figure S8: Effect of the extracorporeal bypass on cardiovascular parameters for the LAD with a microchannel height of 130 µm: (a) blood flow rate while the LAD was using oxygen as the sweep gas, (b) heart rate while the LAD was using oxygen as the sweep gas, (c) mean LAD/shortcut pressure while the LAD was using oxygen as the sweep gas, (d) mean systemic arterial blood pressure measured at the femoral artery while the LAD was using oxygen as the sweep gas, and (e) pulse oximeter saturation level.

Gas exchange parameters for the LAD with a microchannel height of 130 µm and the piglet

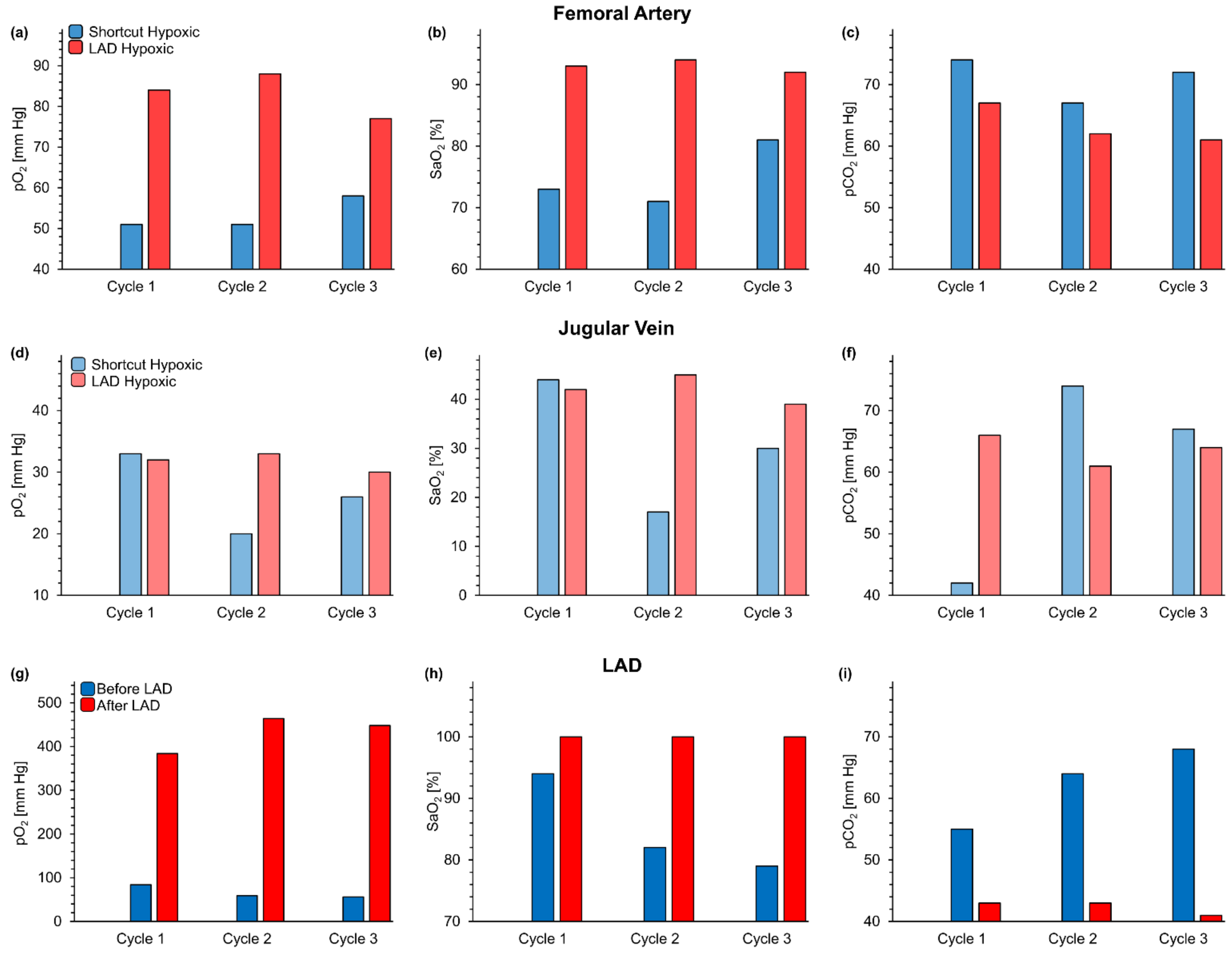

Figure S9: Gas exchange at the femoral artery, jugular vein, and the LAD with a microchannel height of 130 µm when connected in-vivo to a piglet. Measurements were of the blood at the inlet and outlet of the LAD when it was connected to the piglet and pumped by the arterio-venous pressure difference: (a) pO_2,(b) Sa O_2, (c) pCO_2 at the femoral artery, (d) pO_2, (e) Sa O_2, (f) pCO_2 at the jugular vein, and (g) pO_2,(h) Sa O_2, (i) pCO_2 before and after the LAD. Pure oxygen was used as the sweep gas.

Metabolic analysis by sampling from the femoral artery for the LAD with a microchannel height of 130 µm and the piglet

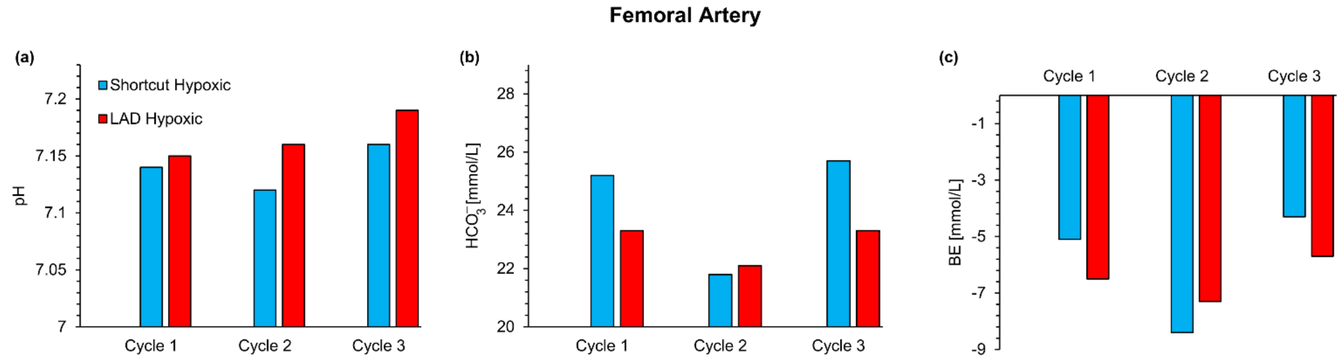

Figure S10: The metabolic analysis: (a) pH measurement, (b) bicarbonate content, and (c) base excess pCO_2 for the femoral artery using oxygen as the sweep gas.

Photos of the in-vivo experimental setup

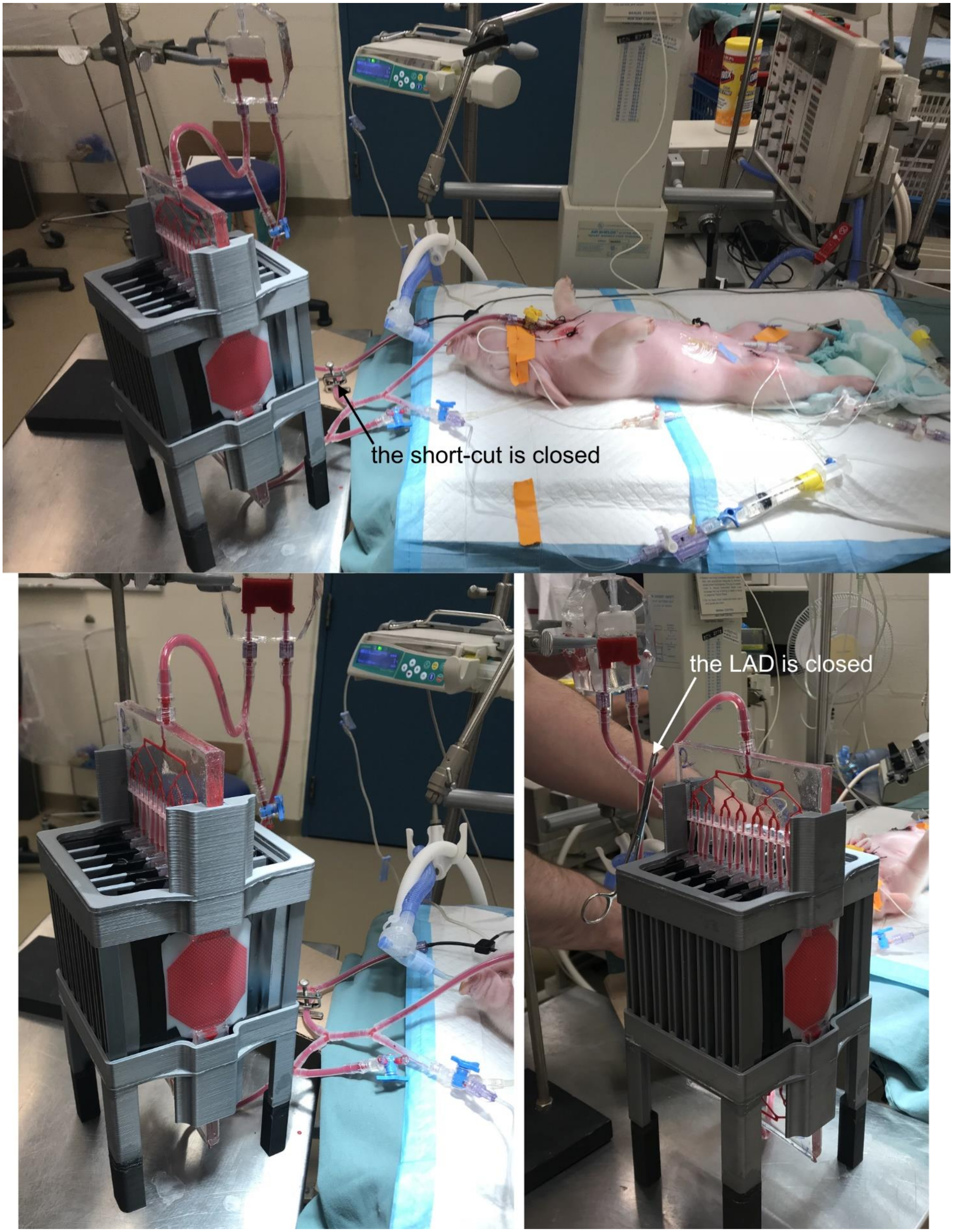

Figure S11: photos from the animal experiments.

References

[1] M. Dabaghi, N. Saraei, G. Fusch, N. Rochow, J. L. Brash, C. Fusch, P. R. Selvaganapathy, *Lab Chip* **2018**, *18*, 3780.

[2] M. Dabaghi, N. Saraei, G. Fusch, N. Rochow, J. L. Brash, C. Fusch, P. R. Selvaganapathy, *J. Memb. Sci.* **2020**, *596*, 117741.

[3] N. Rochow, A. Manan, W.-I. Wu, G. Fusch, S. Monkman, J. Leung, E. Chan, D. Nagpal, D. Predescu, J. Brash, P. R. Selvaganapathy, C. Fusch, *Artif. Organs* **2014**, *38*, 856.

[4] M. Dabaghi, N. Saraei, G. Fusch, N. Rochow, J. L. Brash, C. Fusch, P. Ravi Selvaganapathy, *Biomicrofluidics* **2019**, *13*, 034116.

[5] M. Dabaghi, G. Fusch, N. Saraei, N. Rochow, J. l Brash, C. Fusch, P. R. Selvaganapathy, *Biomicrofluidics* **2018**, *12*, 044101.
